# Plant Cell Wall Plasticity under Stress Situations

**DOI:** 10.3390/plants11202752

**Published:** 2022-10-18

**Authors:** Penélope García-Angulo, Asier Largo-Gosens

**Affiliations:** 1 Faculty of Biological and Environmental Sciences, Universidad de León, 24007 Leon, Spain; 2 Centro de Biotecnología Vegetal, Universidad Andrés Bello, Santiago 8370186, Chile

This Special Issue, entitled “Plant Cell Wall Plasticity under Stress Situations”, is a compilation of five articles, whose authors deepen our understanding of the roles of different cell wall components under biotic and abiotic stress.

The plant cell wall is mainly formed of complex polysaccharides, with multiple interactions between the components that form a network which must be extensible, so as to enable cell expansion, rigid, so as to resist compression and tension forces, and modifiable in response to environmental changes.

Cellulose, the most abundant and resistant polysaccharide on earth, is the main component of the cell wall. The cellulose scaffold is involved in a matrix formed of polysaccharides, such as pectins and hemicelluloses, whose types and proportions vary depending on the species, tissue, and cell type. The deposition of lignin—the second most abundant polymer on earth—in secondary cell walls increases the resistance, leading to growth cessation. All these polymers are crosslinked into the wall in a process that can occur spontaneously and/or by the actions of different modifying enzymes. The control of the synthesis of these cell wall components and/or the interactions between them gives this structure a high plasticity, which is a key factor in the modulation of growth and defense responses under different types of stress.

The evolution of the cell wall allows plants to colonize the land, acquiring the typical erect appearance that allows them to grow taller in order to ensure the absorption of sunlight. Among other important functions, the transport of water and nutrients throughout the plant would not be possible without lignin, which enables the cell wall to be adequately resistant, allowing for long-distance water transport. The deposition and coupling of lignin in the cell wall are controlled by several enzymes, such as laccases and peroxidases. In the first paper of this Special Issue [1], the authors demonstrate the existence of peroxidases with high homology to those of angiosperms in a non-vascular plant, *Physcomitrium patens.* Despite the lack of lignin in this moss, the study describes the discovery of novel class-III peroxidases that could be involved in the biosynthesis of lignin-like polyphenols present in the plant’s primitive water transport system. This discovery could be important for the study of the evolution of lignin biosynthesis in vascular plants.

As we have previously mentioned, the cell wall is involved in the control and uptake of water. The hydration capacity of this structure depends on its composition, which eventually alters the water potential of the cell. In the second paper presented in this Special Issue [2], the authors demonstrate, by means of in vitro experiments with bacterial cellulose composites and plant cell wall components, such as pectins and hemicelluloses, that the water potential and degree of hydration depend on the cell wall composition. The authors also demonstrate that the presence of cell wall proteins facilitates wall rehydration and swelling.

Due to the jellification capacity of pectins, researchers have always considered that they play a key role in cell wall rehydration. In the third paper of this Special Issue [3], based on the performance of in vitro and in vivo experiments, the authors demonstrate that pectin cross-linking modifications involving calcium ions and/or boric acid alter the viscosity of pure pectin standards and prevent their water loss. These modifications affect not only water retention in the cell wall but also its defense capacity against fungal pathogens. In line with this study, the fourth paper [4] demonstrates how the cell wall composition of *Phaseolus vulgaris* L. affects its enzymatic degradation, which ultimately has an effect on the host’s susceptibility to *Pseudomonas syringae* pv. *phaseolicola*. Interestingly, cell wall remodeling can be induced artificially by means of priming molecules which increase the resistance of crops to diseases.

Plants modify their cell walls not only during growth and development but also when they are under attack, and several enzymes are involved in this remodeling. In the fifth paper included in this Special Issue [5], the authors review the latest knowledge regarding the roles of plant β-glucanases in the remodeling and turnover of cell walls, as well as in plant defense and adaptative responses.

The cell wall is a resistant but extremely dynamic cell compartment which controls multiple functions, such as defense responses, growth control, water retention, and ion and molecule exchange, among others. Due to its great diversity in terms of its components and interactions, the cell wall has a wide variety of physicochemical and mechanical properties, which means that this structure is very interesting with respect to the development several useful applications. For instance, we wear cell walls, write on cell walls, eat and feed on cell walls, produce heat by burning cell walls, and we can even build housing made of cell walls. With this Special Issue, we encourage the further study of this important and fascinating structure, and we hope that the reader enjoys and learns from reading it.
